# Characterization of novel cold-active chitin deacetylase for green production of bioactive chitosan

**DOI:** 10.1186/s13568-024-01804-2

**Published:** 2025-01-04

**Authors:** Mohamed N. Abd El-Ghany, Salwa A. Hamdi, Ahmed K. Zahran, Mustafa A. Abou-Taleb, Abdallah M. Heikel, Muhammed T. Abou El-Kheir, Mohamed G. Farahat

**Affiliations:** 1 Botany and Microbiology Department, Faculty of Science, Cairo University, Giza, 12613 Egypt; 2 Zoology Department, Faculty of Science, Cairo University, Giza, 12613 Egypt; 3 Biotechnology / Molecular Biochemistry Program, Faculty of Science, Cairo University, Giza, 12613 Egypt; 4 Biotechnology Department, Faculty of Nanotechnology for Postgraduate Studies, Cairo University, Sheikh Zayed Branch Campus, Giza, 12588 Egypt

**Keywords:** Chitin extraction, Chitin deacetylase, Psychrophilic bacteria, Antifungal chitosan, Spore germination, Membrane permeability, ROS

## Abstract

A Novel cold-active chitin deacetylase from *Shewanella psychrophila* WP2 (SpsCDA) was overexpressed in *Escherichia coli* BL21 and employed for deacetylation of chitin to chitosan. The produced chitosan was characterized, and its antifungal activity was investigated against *Fusarium oxysporum*. The purified recombinant SpsCDA appeared as a single band on SDS-PAGE at approximately 60 kDa, and its specific activity was 92 U/mg. The optimum temperature and pH of SpsCDA were 15 °C and 8.0, respectively, and the enzyme activity was significantly enhanced in the presence of NaCl. The bioconversion of chitin to chitosan by SpsCDA was accomplished in 72 h, and the chitosan yield was 69.2%. The solubility of chitosan was estimated to be 73.4%, and the degree of deacetylation was 78.1%. The estimated molecular weight of the produced chitosan was 224.7 ± 8.4 kDa with a crystallinity index (CrI) value of 18.75. Moreover, FTIR and XRD spectra revealed the characteristic peaks for enzymatically produced chitosan compared with standard chitosan, indicating their structural similarity. The produced chitosan inhibited spore germination of *F. oxysporum* with a minimum inhibitory concentration (MIC) of 1.56 mg/mL. The potential antifungal effect of chitosan is attributed to the inhibition of spore germination accompanied by ultrastructural damage of membranes and leakage of cellular components, as evidenced by transmission electron microscopy (TEM), and accumulation of reactive oxygen species (ROS) that was confirmed by fluorescence microscopy. This study shed light on the cold-active chitin deacetylase from *S. psychrophila* and provides a candidate enzyme for the green preparation of chitosan.

## Introduction

Chitin, β-(1–4) N-acetyl-d-glucosamine, is the second most prevalent biopolymer on the Earth (Jiménez-Gómez and Cecilia 2020). Though crustaceans are the common source, chitin can be extracted from insects, mushrooms, and certain fungi (Liao and Huang 2020; Salehinik et al. 2021; Alimi et al. 2023; Chalghaf et al. 2023; Machado et al. 2024). Despite its abundance, chitin is an undesirable choice in industry owing to its crystallinity and insolubility. This drawback is addressed by modifying chitin to improve its hydrophilicity and reduce the crystallinity. Hence, its soluble deacetylated derivative (chitosan) is gaining importance in food (Liu et al. 2024), medicine (Sharma 2024), industry (Pawariya et al. 2024), environmental protection (Fila et al. 2024), and agriculture through fibers, gels, films, sponges, nanoparticles, and beads (Das et al. 2024; El-Araby et al. 2023; Saberi Riseh et al. 2024). Chitosan can be produced from chitin through deacetylation (Aranaz et al. 2021). Unlike chitin, chitosan is soluble in aqueous acidic solutions via primary amine protonation, and its solubility depends on the degree of acetylation (Zargar et al. 2015). Chitosan is a superior candidate for a wide array of applications owing to its macromolecular structure and unique properties, including solubility, biocompatibility, biodegradability, and reactivity (Thambiliyagodage et al. 2023). It can be used in the purification of wastewater for the removal of heavy metal ions (Karimi et al. 2022), dyes (Salman et al. 2023), and pesticides (Altun et al. 2023; Fu et al. 2023). Moreover, it has numerous medical applications such as drug delivery (Kumar et al.), wound dressing (Gupta and Vyas 2023; Zhao et al. 2023), tissue engineering (Zhang et al. 2023a), dentistry (Arora et al. 2023; Paradowska-Stolarz et al. 2023), and ophthalmology (Popova et al. 2022; Tan et al. 2023). On this point, the antitumor effect of chitosan and its derivatives has been explored, and literature has pointed out their direct impact on the cancer itself in addition to immunomodulation (Han et al. 2016; Maleki Dana et al. 2021; Lima et al. 2021; Zaiki et al. 2023). Besides, literature explored many biological activities of chitosan and its derivatives, such as antioxidant, anti-inflammatory, and antimicrobial activities (Abd El-Hack et al. 2020; Yang et al. 2023).

Mainly, chitosan is produced by the deacetylation of chitin through concentrated alkali treatment at elevated temperatures, yet this chemical route used for chitin deacetylation typically involves harsh chemicals that cause environmental pollution (Kozma et al. 2022; Islam et al. 2022). Alternatively, chitin deacetylase (CDA) can be used as an eco-friendly biological route to produce chitosan from chitin (Roman et al. 2019). Chitin deacetylases (EC 3.5.1.41) are enzymes that can catalyze the de-N-acetylation process of chitin (break down chitin's acetamido group to produce chitosan through hydrolysis), and the majority of recognized CDAs possess the conserved NodB homology domain or polysaccharide deacetylase catalytic domain (Huang et al. 2022). So far, the literature covers the description of various CDAs from bacterial and fungal origins such as *Acinetobacter schindleri* (Yang et al. 2022b), *Bacillus cereus* (Zhang et al. 2023b), *Streptomyces bacillaris* (Yin et al. 2023), *Streptomyces diastaticus* (Xu et al. 2020), *Cryptococcus neoformans* (Lee et al. 2020), *Pochonia chlamydosporia* (Aranda-Martinez et al. 2018), and other sources. Hitherto, the most reported CDAs are derived from mesophilic or thermophilic microorganisms operated at relatively elevated temperatures, and there is limited published data on cold-active CDAs derived from psychrophilic origin. In this investigation, we address a novel cold-active CDA from the psychrophilic bacterium, *S. psychrophila,* as a potent candidate for green preparation of chitosan at low temperature. The produced chitosan was characterized, and its antifungal activity was evaluated against *F. oxysporum* with emphasis on the potential antifungal mechanism.

## Materials and methods

### Sequence analysis and construction of SpsCDA expression vector

The amino acid sequence of SpsCDA from *S. psychrophila* WP2 was retrieved from GenBank (Accession number: AQS40340.1). The existence of a signal peptide was predicted using SignalP 5.0 server (http://www.cbs.dtu.dk/services/SignalP). Furthermore, the interProScan and ProtParam tools were utilized to classify and predict the physicochemical properties of the protein (Shehata et al. 2023). Prosite database (https://prosite.expasy.org/scanprosite/) was used for analyzing the motif and functional sites. The gene sequence of SpsCDA, excluding its native signal sequence, was codon-optimized and synthesized by NovoPro Bioscience Inc. (Minhang District, Shanghai, China). The nucleotide sequence of the codon-optimized synthetic construct (1527 bp) was submitted to the GenBank (accession number OR762151). Multiple sequence alignment analysis of the deduced amino acids was performed using Clustalw online tool (https://www.genome.jp/tools-bin/clustalw) and visualized using CLC Main Workbench software (Version 6.5). The phylogenetic tree was constructed using the neighbor-joining (NJ) method of the MEGA 11 software. The synthesized gene was inserted between the BamHI and HindIII restriction sites of the pQE-80L expression vector (Qiagen, Hilden, Germany). Following the manufacturer's instructions, the resultant plasmid, designated pQE-80L-SpsCDA, was transformed into *E. coli* BL21 (DE3) competent cells using a MicroPulser electroporator (Bio-Rad, Hercules, CA, USA). After electroporation, the transformation mixtures were plated on LB agar plates supplemented with ampicillin (100 µg/mL). Following an 18-h incubation period at 37 °C, the developed colonies (transformants) were picked for further investigations.

### Expression and characterization of SpsCDA

The transformant cells were cultured in LB broth, containing 100 μg/mL ampicillin, with shaking (180 rpm) at 37 °C until reaching an OD_600_ nm of 0.6. Afterwards, IPTG was added at a final concentration of 1 mM to induce the expression, and the induced cells were harvested by centrifugation 4h later. Then, the collected cells were resuspended in a lysis buffer (50 mM NaH_2_PO_4_, 300 mM NaCl, 10 mM imidazole, pH 8.0) containing lysozyme (1 mg/mL) and subjected to sonication. Subsequently, the His-tagged SpsCDA was purified using a Ni–NTA superflow column (Qiagen, Hilden, Germany), following previously published method (Farahat et al. 2020a). The homogeneity and molecular weight of the recombinant SpsCDA was analyzed by 12% SDS-PAGE. The Bradford method was used to estimate the protein concentration (Bradford 1976). The chitin deacetylase activity (enzyme assay) of SpsCDA was determined using 4-nitroacetanilide as a substrate (Sun et al. 2014). Briefly, the reaction mixture containing 100 µL SpsCDA, 3 mL phosphate buffer (100 mM, pH 7.0), and 1 mL 4-nitroacetanilide (200 μg/mL) was incubated at 15 °C for 60 min. Subsequently, the reaction was then terminated by heating in a boiling water bath for 3 min. Following centrifugation at 8,000 × g for 10 min, the release of 4-nitroaniline was determined by measuring the absorbance of the supernatant at 400 nm using a double beam UV–Vis spectrophotometer (UV-1800, Shimadzu, Japan). One unit of chitin deacetylase is defined as the amount of enzyme that catalyzes the release of 1 μg of 4-nitroaniline per hour from 4-nitroacetanilide. To determine the optimum temperature, pH and salt concentration for SpsCDA activity, the above-described enzyme assay was run at several temperatures, pH values, and NaCl concentrations. The optimum temperature of SpsCDA was inspected by assaying its activity at various temperatures (5–50 °C). The optimal pH of SpsCDA was determined by conducting the enzyme assay with different pH values (3.0–12.0) at the optimum temperature (15 °C) using sodium citrate buffer (pH 3–5), phosphate buffer (pH 6–7), Tris–HCl (pH 8–9), sodium carbonate-sodium hydroxide (pH 10–11), and disodium hydrogen phosphate-sodium hydroxide (pH 12). The influence of different NaCl concentrations up to 2 M on SpsCDA activity was examined at the optimum temperature (15 °C) and pH (8.0). The influence of various metal ions (Zn^2+^, Cu^2+^, Hg^2+^, Mg^2+^, Ni^2+^, Pb^2+^, and Mn^2+^) and EDTA on SpsCDA activity was determined by performing enzyme assay in the presence of 1 mM of the corresponding metal-salts or EDTA and expressed as relative activity (%).

### Conversion of chitin to chitosan by SpsCDA

The enzymatic deacetylation of chitin to chitosan by the recombinant SpsCDA was assessed at the optimum conditions. In brief, two grams of chitin powder (from shrimp shells, ≥ 75% deacetylated, Sigma Aldrich) were resuspended in 100 mL of 50 mM Tris–HCl buffer (pH 8.0) containing 0.6 M NaCl. Then, SpsCDA (200 U) was added, and the mixture was incubated at 15 °C for 72 h. After centrifugation at 10,000 × g at 4 °C, the precipitated chitosan was collected, washed with deionized water, and dried. The yield of the produced chitosan was calculated based on its dry weight relative to the dry weight of chitin, as described elsewhere (Rakshit et al. 2023). The solubility analysis was achieved by dissolving chitosan in 1% acetic acid. After centrifugation at 10,000 × g for 10 min, the undissolved solid matter was recovered, dried, and weighed. Then, the solubility was calculated using the equation described by Kumari et al. (2017). The molecular weight of the prepared chitosan was determined from its intrinsic viscosity [ɳ] by dissolving chitosan in 1% acetic acid, and the viscosity measurements were performed at 25 °C using Modular Compact Rheometer MCR 302e (Anton Paar GmbH, Graz, Austria). Accordingly, the molecular weight of chitosan was determined using the Mark-Houwink equation (Ramasamy et al. 2017). The deacetylation degree (DD) of the prepared chitosan was determined by the acid–base conductometric titration method (Dutta and Priyanka 2022). To confirm the structure of the produced chitosan, its FTIR and XRD spectra were recorded and compared with those of a standard chitosan (Sigma Aldrich). In this regard, FTIR spectroscopy was used to characterize the enzymatically prepared chitosan, and the spectra were recorded using a Nicolet 6700 FT-IR spectrometer (Thermo Scientific, MA, USA) in the range of 4000 to 400/cm. Finally, the prepared chitosan was subjected to the XRD analysis using a D8 Discover diffractometer (Bruker, Karlsruhe, Germany) with Cu Kα radiation (k = 40 kV, 30 mA) between 2θ angles ranging from 5 to 50°. The crystallinity index (CrI) of the chitosan was estimated using the equation: CrI = [(I_110_ − I_am_)/I_110_] × 100; where I_110_ is the intensity of the diffraction peak at 2θ = 20° and I_am_ is the intensity of amorphous diffraction at 2θ = 16° (Le Goff et al. 2020). The scanning electron microscopy was performed to investigate the microstructure of chitin (substrate) and the produced chitosan by using a Scanning Electron Microscope (FEI Quanta FEG 250, FEI Corporation, USA).

### Antifungal activity of chitosan

The antifungal activity of the prepared chitosan was evaluated against *F. oxysporum* AUMC 10313 provided by the Assiut University Mycology Center (AUMC), Assiut, Egypt. The antifungal activity was investigated using a resazurin microtiter plate assay. Succinctly, spores of *F. oxysporum* were collected from cultures that had been growing for seven days on potato dextrose agar (PDA) plates (Merck KgaA, Darmstadt, Germany), and suspended in a sterile saline solution. After filtering the resultant suspension through sterile muslin, the spore count was adjusted to 2 ⨯ 10^6^ spores/mL. Subsequently, 50 µL of the prepared spore suspension was added to each well of a 96-well microtiter plate that contained 150 μL of potato dextrose broth with varying concentrations of chitosan (0.024–50 mg/mL). Then resazurin was added to a final concentration of 0.002% (w/v), and the plates were incubated at 28 °C for 24 h for germination. Also, wells without inoculum were the negative control. After incubation, the color of resazurin was visually inspected and the minimum inhibitory concentration (MIC) was determined. The presence of metabolic activity (spore germination) is indicated by the pink color, whereas the absence of metabolic activity (no spore germination) is indicated by the blue color.

### Impact of chitosan on spore morphology and ultrastructure

The prepared spore suspensions of *F. oxysporum* were exposed to sublethal concentrations (about half of MIC) of chitosan. Control experiments were performed using the same procedure but without addition of chitosan. The spores were collected and fixed after 8 h of incubation at 28 °C, and ultrathin sections were prepared as described elsewhere (Abd El-Ghany et al. 2023b). Finally, the ultrathin sections were observed by transmission electron microscopy (TEM) at 80 kV using a JEM-1400 transmission electron microscope (JEOL, Tokyo, Japan).

### Leakage of proteins and DNA

The impact of chitosan on the membrane integrity of *F. oxysporum* was assessed by measuring the amount of proteins and DNA that leaked from exposed phytopathogen. Practically, spore suspensions of *F. oxysporum* were incubated for 24 h at 28 °C with chitosan at the half MIC value. Subsequently, cultures were centrifuged at 10,000 × g for 15 min at 4 °C, and the protein and DNA contents were determined in the supernatant by a NanoDrop2000 spectrophotometer (Thermo Fisher Scientific, MA, USA) at 280 and 260 nm, respectively, and compared with those of the untreated spore suspension sets (control).

### Analysis of reactive oxygen species (ROS)

The accumulation of ROS induced by the exposure of *F. oxysporum* spores to chitosan was assessed by the fluorescent dye 2′,7′-dichlorofluorescein diacetate (DCFH-DA) (Hernández-Téllez et al. 2022). Briefly, 100 µL of the *F. oxysporum* spore suspension (2 × 10^6^ spores/mL) was added to each well in a 96-well microplate. Then, chitosan was added at a concentration of half the MIC value. At the same time, wells without adding chitosan were considered as control. After incubation at 28 °C for 4 h, the spores were stained with DCFH-DA solution, incubated at 28 °C for 30 min, and visualized using an EVOS™ M5000 inverted fluorescence microscope (Thermo Fisher Scientific, MA, USA).

### Statistical analysis

In this study, the analysis of variance was performed on the measured data (ANOVA) using IBM SPSS software version 22. The significant differences between treatments were compared with the critical difference at the 5% level of probability using Duncan's test.

## Results

### Sequence analysis, expression and characterization of SpsCDA

**Fig. 1:**
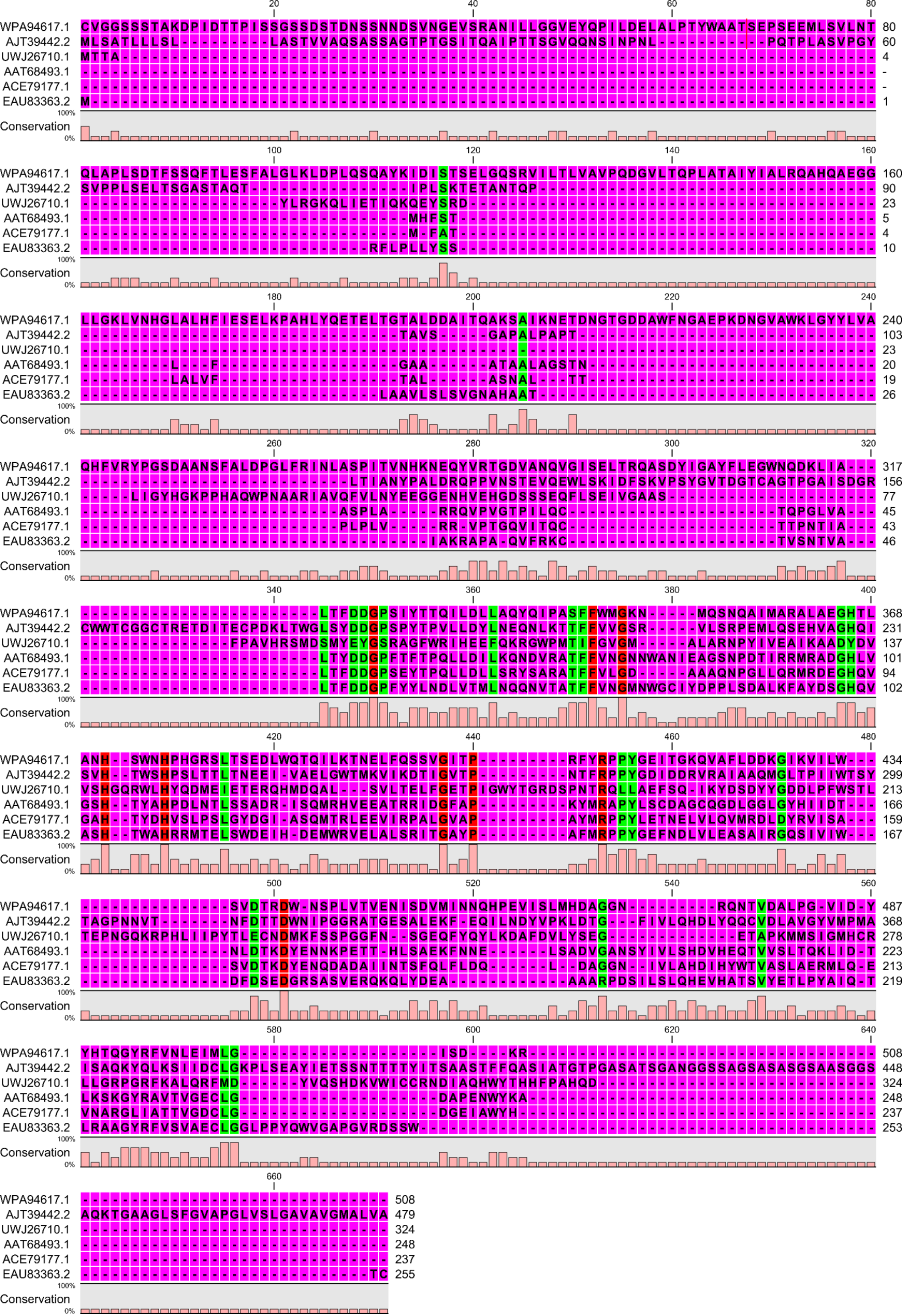
Multiple sequence alignment of of SpsCDA derived from *S. psychrophila* with that of previoulsy reported chitin deacetylases. Green columns indicated highly conserved amino acid residues while red columns indicated completely consirved residues

**Fig. 2:**
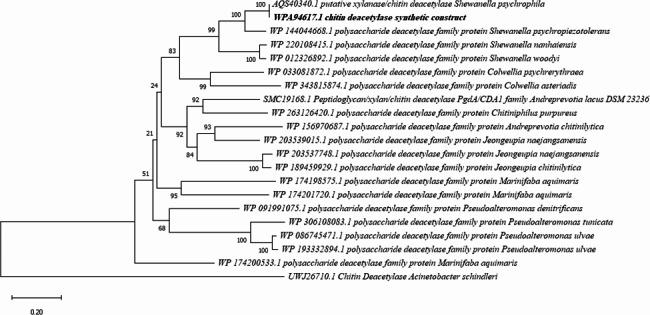
Phylogenetic tree depicted the degree of relatedness among SpsCDA derived from *S. psychrophila* to other bacterial chitin deacetylases

The amino acid sequence of SpsCDA from *S. psychrophila* WP2 was retrieved from GenBank. Based on results obtained from the in-silico characterization, the protein sequence is predicted to comprise 545 amino acids (aa) with an N-terminal signal peptide of 37 residues. InterProScan results predicted that the protein belongs to the Glycoside hydrolase/deacetylase, beta/alpha-barrel superfamily (IPR011330) and indicated the presence of NodB homology domain (IPR002509) chitooligosaccharide deacetylase. Likewise, PROSITE analysis revealed that SpsCDA contains NodB homology domain profile (PS51677), and the active site residue is predicted to be the amino acid H505. Regarding the physicochemical properties of the mature sequence of SpsCDA (without its native signal peptide) predicted by Protparam, the enzyme was classified as a stable protein; with an instability index (II) of 33.5. The protein's computed molecular weight was 55.65 kDa, and its GRAVY value was −0.245. The calculated aliphatic index and theoretical pI values were 90.28 and 4.56, respectively. Multiple sequence alignment analysis of the deduced amino acid sequence (508 aa) of *S. psychrophila* WP2 chitin deacetylase (SpsCDA, GenBank: WPA94617.1) with previously reported chitin deacetylases derived from *Acinetobacter schindleri* (GenBank: UWJ26710.1) (Yang et al. 2022b), *Cryptococcus laurentii* (GenBank: AJT39442.2) (Chakraborty et al. 2016), *Colletotrichum lindemuthianum* (GenBank: AAT68493.1) (Kang et al. 2012), *Aspergillus nidulans* (GenBank: ACE79177.1) (Wang et al. 2010), and *Coprinopsis cinerea* (GenBank: EAU83363.2) displayed 29 highly conserved residues, among them nine completely conserved amino acid sites (Fig. 1). The neighbor-joining phylogenetic tree depicted the degree of relatedness among SpsCDA and other chitin deacetylase from a wide variety of bacterial species (Fig. 2). Consequently, the codons of SpsCDA were optimized in accordance with the codon bias of *E. coli*, and the gene was synthesized. The synthetic gene (without its native signal peptide sequence) was inserted downstream of the N-terminal histidine tag of the pQE-80L expression vector and transformed into *E. coli* BL21 (DE3).

**Fig. 3:**
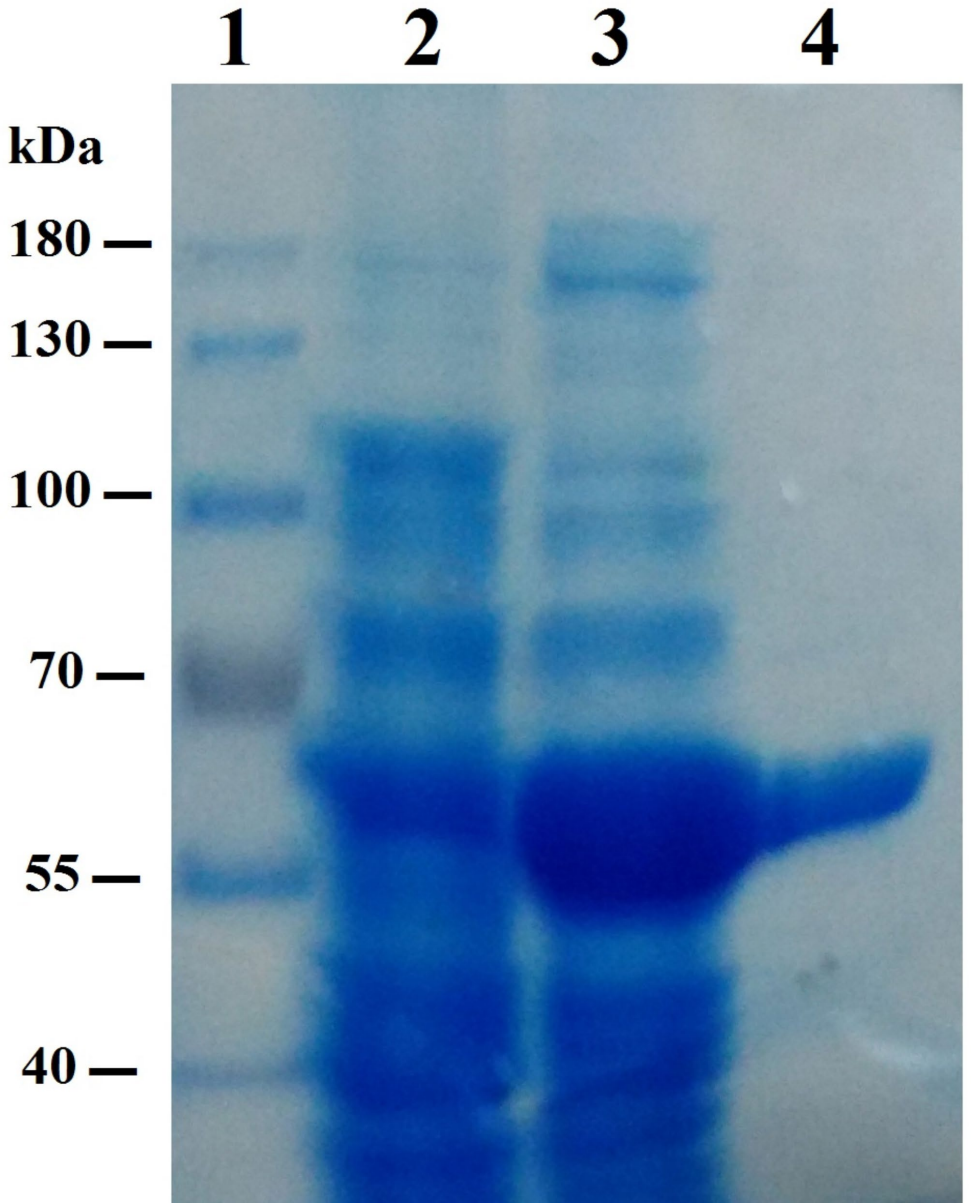
SDS-PAGE analysis of the expression and purification of SpsCDA derived from *S. psychrophila*. Lane 1 indicates the molecular weight marker. Lanes 2, 3 and 4 stand for total soluble proteins of uninduced *E. coli* BL21, total soluble proteins of IPTG-induced *E. coli* BL21, and Ni–NTA-purified His-tagged SpsCDA (≈60 kDa), respectively

**Fig. 4:**
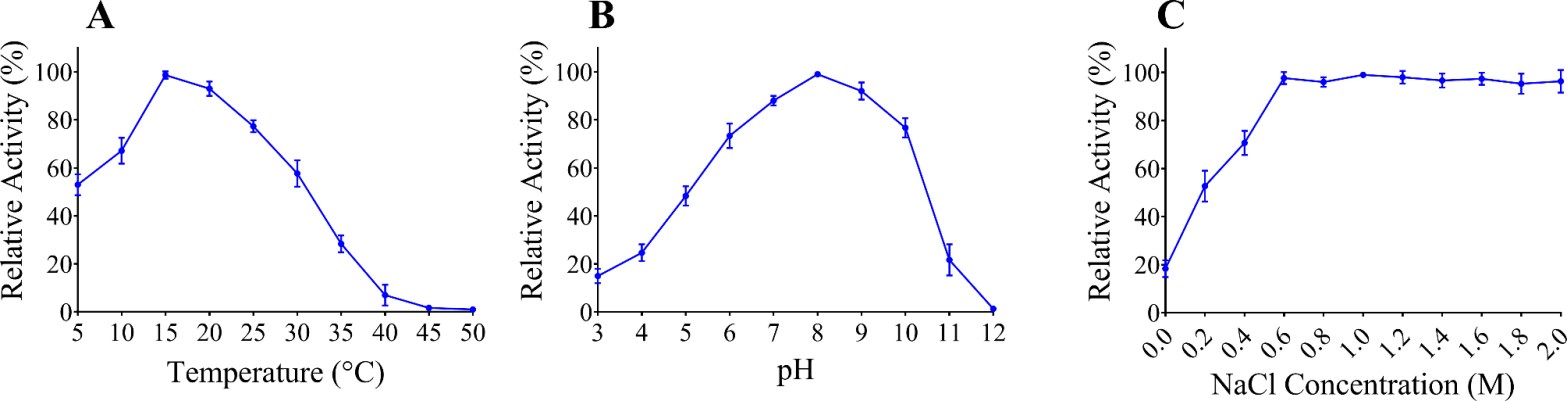
Effect of temperature (**A**), pH (**B**), and NaCl concentration (**C**) on SpsCDA activity. The presented data are the means of triplicate assays. Error bars indicate the standard deviation (SD)

The affinity chromatography was utilized to purify the recombinant SpsCDA protein by using a Ni–NTA resin column and the SDS-PAGE analysis verified its purity as a single protein band at approximately 60 kDa (Fig. 3). Purified SpsCDA was produced as an active and soluble enzyme with a specific activity of 92 U/mg, according to the deacetylation assay using 4-nitroacetanilide as a substrate. Results revealed that SpsCDA was a cold-active enzyme, with an optimum temperature of 15 °C, that exhibited more than 50% of its maximum activity at 5 °C, and complete inhibition was observed at 45 °C (Fig. 4A). The optimum pH was estimated to be 8.0 in 50 mM Tris–HCl buffer (Fig. 4B). Increasing the salt concentration caused a dramatic increase in enzyme activity up to 600 mM NaCl; further increase in NaCl concentration resulted in the flattening of the curve up to 2M NaCl (Fig. 4C). Regarding the effect of metal ions, a remarkable enhancement of the enzymatic activity was observed in the presence of Zn^2+^ (188%) and Ni^2+^ (175%). Also, the exposure of SpsCDA to Cu^2+^, Mg^2+^, and Mn^2+^ boosted the enzymatic activity by 117, 129 and 138%, respectively. No obvious impact was recorded in the presence of Hg^2+^ and Pb^2+^. Conversely, EDTA exerted significant inhibitory impact on SpsCDA (Table 1).

**Table 1:** Influence of different chemical ions and EDTA on SpsCDA activity

Chemical effector	Relative activity (%)
Control	100^d^
Zn^2+^	188.2 ± 8.4^a^
Cu^2+^	117.1 ± 9.7^c^
Hg^2+^	94.9 ± 8. 2^d^
Mg^2+^	129.2 ± 6.8^b,c^
Ni^2+^	175.6 ± 9.8^a^
Pb^2+^	99.4 ± 7.5^d^
Mn^2+^	138.5 ± 9.2^b^
EDTA	28.2 ± 3.6^e^

The same letter indicates that there is an insignificant difference between these treated groups according to Duncan’s multiple range test (p < 0.05)

### Conversion of chitin to chitosan by SpsCDA

**Fig. 5:**
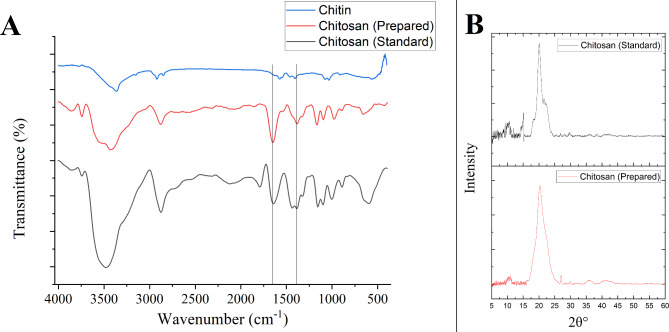
FTIR (**A**) and XRD (**B**) spectra of the prepared chitosan and standard chitosan

**Fig. 6:**
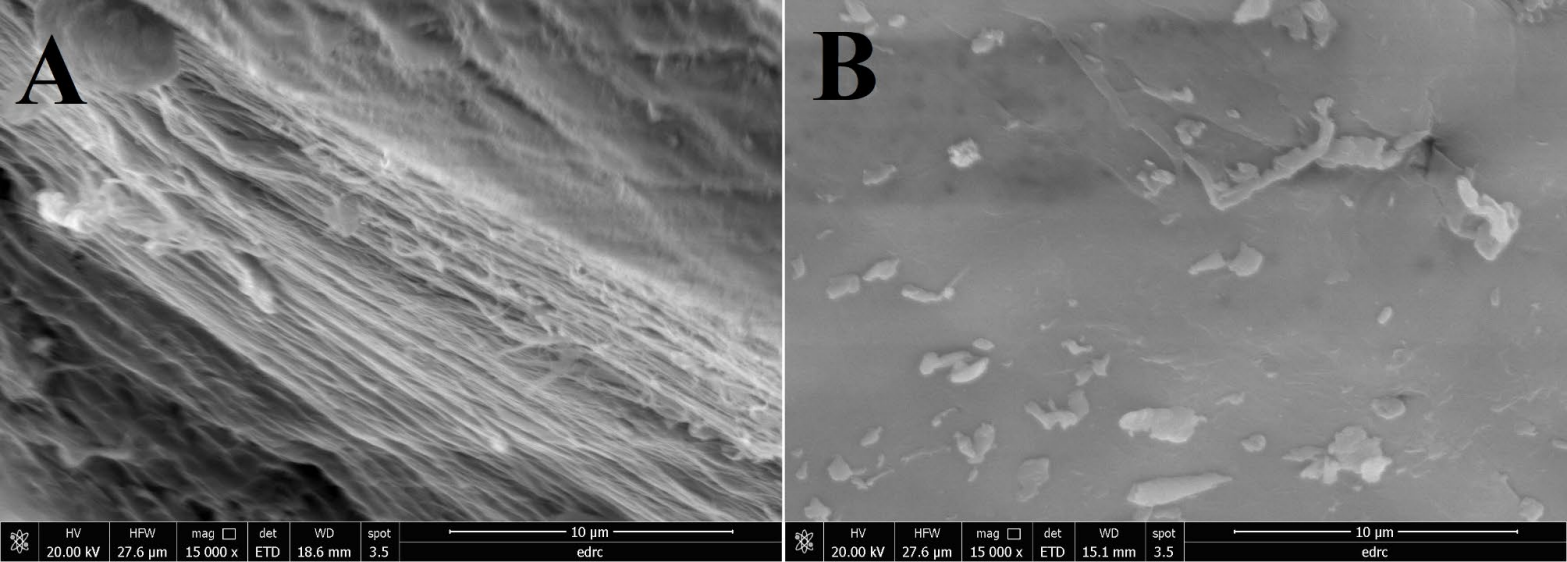
SEM images of chitin (**A**) and chitosan (**B**)

Based on the optimum conditions at which the maximum enzyme activity was achieved, the subsequent chitin deacetylation experiments were carried out at 15 °C in a reaction buffer containing 600 mM NaCl at pH 8.0. The bioconversion of chitin to chitosan by the purified SpsCDA was accomplished in 72 h, and the chitosan yield was 69.2 ± 1.2%. The solubility of the enzymatically produced chitosan was estimated to be 73.4 ± 1.7%, and the degree of deacetylation was found to be 78.1 ± 2.3% (The degree of deacetylation of the standard chitosan was estimated to be 83.6 ± 1.1%). The estimated molecular weight of produced chitosan was 224.7 ± 8.4 kDa. The chemical structure of the chitosan produced by SpsCDA was investigated by FTIR spectroscopy and compared with that of standard chitosan and chitin (Fig. 5A). Furthermore, the XRD spectra of the produced chitosan showed two major peaks at around 10 and 20° implying structural resemblance to the standard chitosan (Fig. 5B). As deduced from the XRD spectrum, the extracted chitosan had a crystallinity index (CrI) value of 18.75. In addition, SEM investigated the variation of microstructures of chitin and the produced chitosan. Results showed that untreated chitin was stacked in a layered structure whose surface was compactly arranged with a crystalline microfibril structure (Fig. 6A). On the other hand, the fibers of the produced chitosan became blurred and indistinct, exhibiting a less well-defined interface (Fig. 6B).

### Antifungal activity of chitosan

**Fig. 7:**
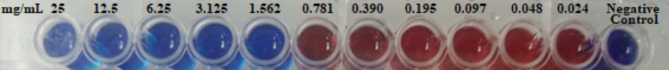
Antifungal activity of chitosan using resazurin microtiter plate assay

The antifungal activity of chitosan was investigated against *F. oxysporum* AUMC 10313 by evaluation of its influence on spore germination using a resazurin microtiter plate assay. Chitosan exhibited a promising inhibitory effect on spore germination of *F. oxysporum*. The MIC of chitosan was 1.56 mg/mL (Fig. 7).

### Impact of chitosan on spore morphology and ultrastructure

**Fig. 8:**
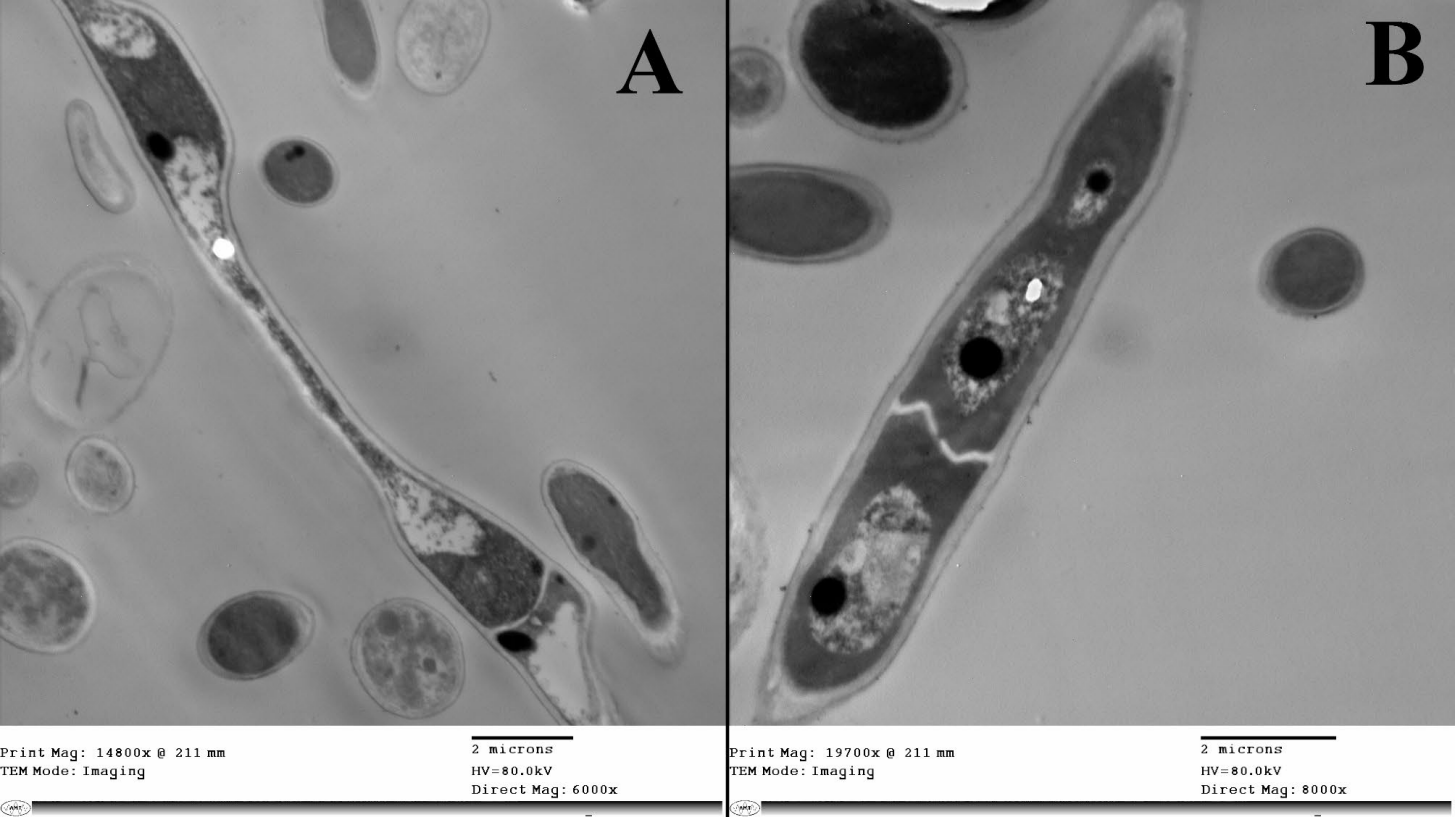
TEM micrographs of *F. oxysporum* spores indicating the ultrastructure alterations. *F. oxysporum* spores upon exposure to the chitosan produced by SpsCDA (**A**), Untreated control (**B**)

Observations by the TEM showed pronounced morphological and ultrastructural alterations in spores of *F. oxysporum* treated with chitosan (0.8 mg/mL). Remarkable loss of cellular materials was observed accompanied by separation of the plasmalemma from the cell wall, and the cytosol of numerous spores seemed empty (Fig. 8A). On the other hand, untreated pores appeared normal, containing a well-defined cell wall and a dense cytoplasm with intact structure (Fig. 8B).

### Leakage of proteins and DNA

**Fig. 9:**
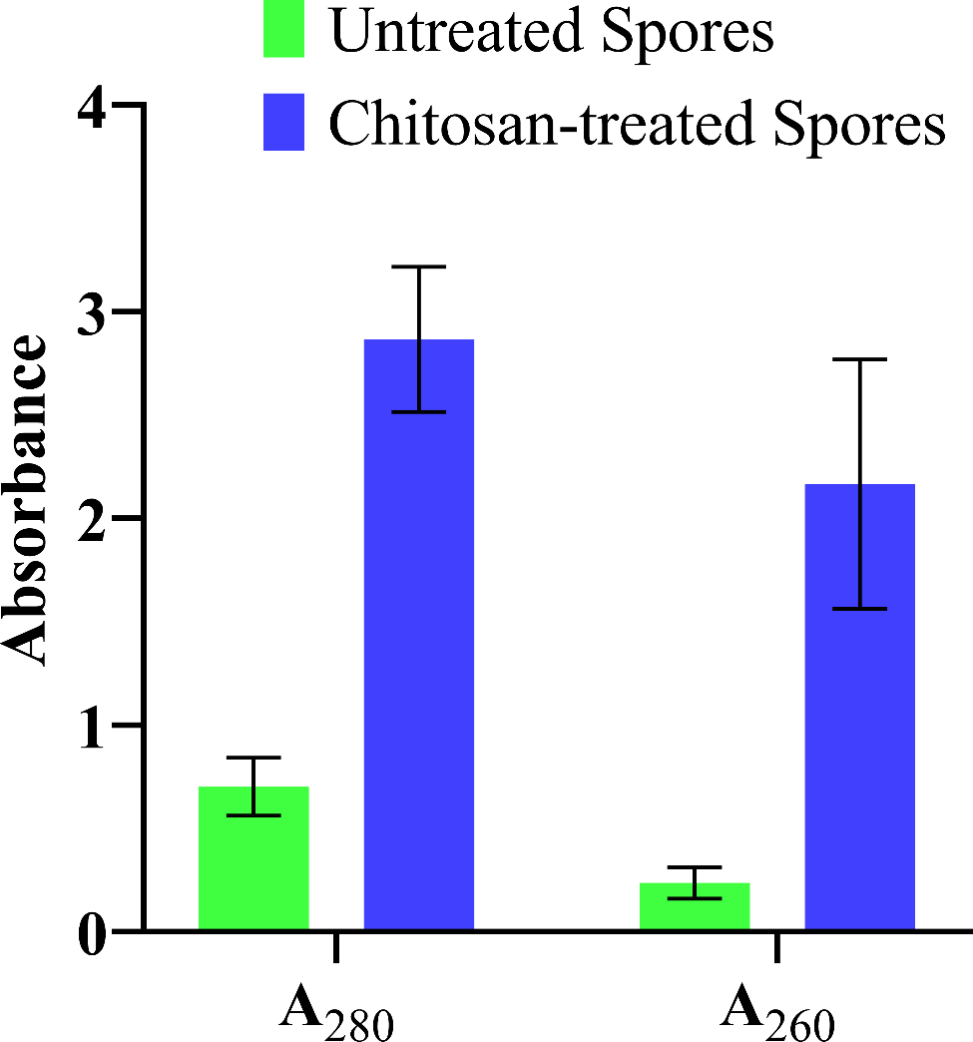
Effect of the chitosan produced by SpsCDA on the leakage of proteins and DNA from spore suspension of *F. oxysporum*

The mechanism behind the antifungal activity of chitosan treatment was also determined by measuring cellular leakages in terms of proteins and DNA. The membrane leakage assays affirmed the membrane damage upon exposure of *F. oxysporum* spores to chitosan. The results indicated a noticeably greater leakage of proteins and nucleic acids than that in the control group (Fig. 9).

### Intracellular accumulation of ROS

**Fig. 10:**
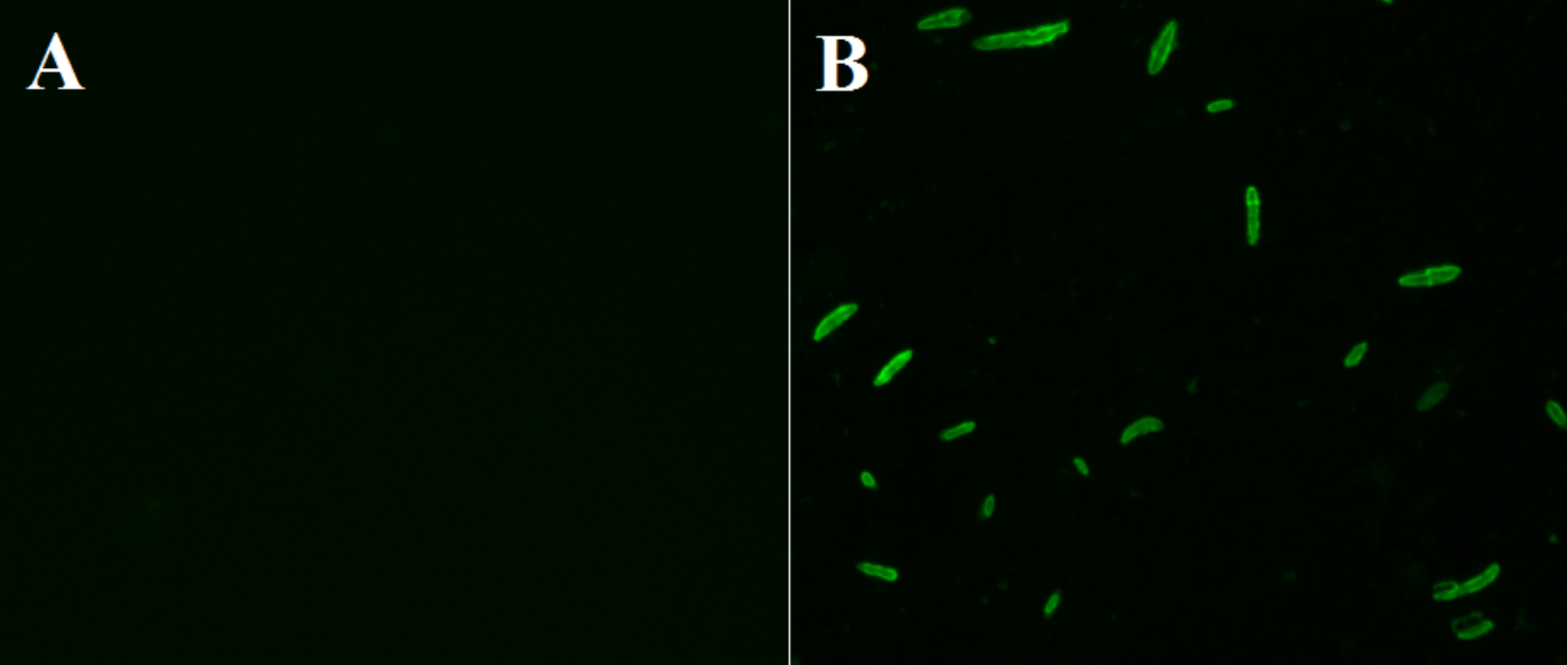
Fluorescence microscope images of ROS accumulation in *F. oxysporum* spores. Untreated spores (**A**), spores exposed to chitosan (**B**)

To investigate the potential involvement of ROS in the damage of chitosan-treated spores, the fluorescence probe DCFH-DA was used to detect the intracellular accumulation of ROS. The fluorescence intensity of the untreated spores was weak (Fig. 10A), whereas the spores treated with chitosan (0.8 mg/mL) produced noticeable green fluorescence, indicating the accumulation of ROS (Fig. 10B).

## Discussion

The deacetylation of chitin to obtain chitosan improves its solubility, thus enhancing the biological properties. Consequently, we developed an eco-friendly approach to achieve the deacetylation of chitosan at low temperatures utilizing a novel chitin deacetylase derived from *S. psychrophila*. As a psychrophilic bacterium, it has been suggested to produce cold-active enzymes (Xiao et al. 2007). After bioinformatics analysis and in silico characterization that predicted the function and structure of SpsCDA, the synthetic codon-optimized sequence of SpsCDA was efficiently over-expressed in *E. coli* BL21 (DE3). It has been widely accepted that codon optimization enhances the heterologous expression of proteins by avoiding codon bias of the host (Farahat et al. 2020b). The over-expressed protein was purified to homogeneity with an apparent molecular weight of 60 kDa. In this respect, most addressed chitin deacetylases derived from bacterial sources had a lower molecular weight than SpsCDA. The molecular weights of chitin deacetylase derived from *Microbacterium esteraromaticum* and *Nitratireductor aquimarinus* MCDA3-3 were approximately 26 and 36 kDa, respectively (Chai et al. 2020; Yang et al. 2022a). Notwithstanding, the molecular weight of chitin deacetylase derived from the marine *Alcaligenes faecalis* was determined to be 66 kDa (Amer et al. 2022). With a specific activity of 92 U/mg, the recombinant SpsCDA was expressed as an active enzyme. This value exceeds that of various reported chitin deacetylases. It has been reported that the purified chitin deacetylase from *N. aquimarinus* (NaCDA) showed 50 U/mg specific activity (Chai et al. 2020). In a similar study, chitin deacetylase from *Bacillus aryabhattai* (BaCDA) showed a specific activity of 38.89 U/mg (Pawaskar et al. 2021). The specific activity of chitin deacetylase obtained from *Streptomyces griseoincarnatus* RB7AG was 3.09 U/mg (Behera et al. 2023). However, the specific activity of the purified chitin deacetylase derived from *M. esteraromaticum* (MeCDA) was 137.54 U/mg (Yang et al. 2022a). Unlike the most reported chitin deacetylases, it is worth mentioning that SpsCDA is a cold-active enzyme, and its optimum temperature was 15 °C. Nonetheless, the optimum temperature of various chitin deacetylases ranged from 30 to 60 °C (Martinou et al. 2002; Shrestha et al. 2004; Zhao et al. 2011; Pawaskar et al. 2021; Yin et al. 2023; Cheng et al. 2023). In this regard, psychrophilic organisms thriving in cold ecosystems are modified to retain their active metabolic processes in low temperatures and produce potential cold-active enzymes. This merit makes psychrophilic organisms more appealing and attractive in biotechnology for the production of active enzymes operating at low temperatures, which implies an important feature for energy saving (Hamid et al. 2022; Collins and Feller 2023; Liu et al. 2023). Furthermore, SpsCDA continued to operate in the absence of NaCl, but its maximum catalytic activity rose when salt was present; this attitude is in context with various enzymes derived from halotolerant and marine bacteria (Farahat et al. 2020a; Djelid et al. 2023). In this study, the enzyme activity was activated by various metal ions including Zn^2+^, Ni^2+^, Cu^2+^, Mg^2+^, and Mn^2+^ and significantly inhibited by the ion-chelating agent (EDTA) suggested that the enzyme is a metallo-protein. These results agree with those reported on the metallo-protein chitin deacetylase derived from *Streptomyces griseoinacarnatus* (Behera et al. 2023). In good agreement, chitin deacetylase derived from *Microbacterium esteraromaticum* MCDA02 was strongly inhibited by EDTA (Yang et al. 2022a). However, previous studies pointed out the stimulatory and inhibitory effect of metal ions on various chitin deacetylases which varies according to the protein sequence and the presence of metal binding sites (Wang et al. 2024; Yin et al. 2022).

In this study, the purified SpsCDA showed fair deacetylation activity and converted chitin to chitosan with a yield of 69.2%. Remarkably, FTIR and XRD spectra of the produced chitosan showed good matching with those of the standard chitosan, however, the slight difference in the XRD spectra may be due to the difference of the deacetylation percent of the prepared chitosan compared with the standard which may affect their crystallinity. Compared with chitin (substrate), the chitosan spectrum displayed distinctive peaks around 1,650 cm^−1^ (amide I, indicating the removal of the acetyl group), and 1,380 cm^−1^ (amide II, representing bending -NH_2_) and confirms successful conversion from chitin to chitosan, with the amide group being replaced by an amine group (Hemmami et al. 2024; Qiao et al. 2021). These findings confirmed the structure of the produced chitosan and indicated the successful conversion of chitin to chitosan by the recombinant SpsCDA. Likewise, chitin deacetylases derived from various microbial sources have been used for production of chitosan from chitin via the deacetylation reaction. Our findings are in harmony with those reported for the utilization of chitin deacetylase from *Alcaligens faecalis* for biotransformation of chitin to chitosan with 71% solubility, 74.9% degree of deacetylation, 21.2% CI, and 246.4 kDa molecular weight (Rakshit et al. 2023). In this study, the untreated chitin showed a prominent arranged microfibrillar crystalline structure in SEM which was absent in the produced chitosan. The reason for microstructure changes in chitin upon treatment by the chitin deacetylase may be attributed to the reduction in acetyl group content, leading to the destruction or weakening of the intramolecular and molecular structure (Berger et al. 2014; Yang et al. 2022b).

Phytopathogenic fungi are one of the main factors losing up to twenty percent of the worldwide crop yields yearly, and about ten percent of crops are ruined due to post-harvest fungal infections (Davies et al. 2021). Of these phytopathogens, *F. oxysporum* is a causative agent of wilt disease which is considered a serious and global problem in numerous crop production (Majeed et al. 2024). Particularely, *F. oxysporum* ranks in abundance as the fifth among the most abundant ten fungal plant pathogens causing overwhelming vascular wilt in many plant species (Husaini et al. 2018). Besides being a phytopathogen, *F. oxysporum* had emerged as a human pathogen (Mallik et al. 2020) causing invasive fungal infections, especially, in primary immunodeficiency diseases patients (Abd Elaziz et al. 2020). In this investigation, the produced chitosan demonstrated potent antifungal activity against *F. oxysporum*. The potential mechanism underlying its antifungal activity may operate by the inhibition of spore germination, alternation of spore ultrastructure, and impairing of membrane permeability that leads to the leakage of cellular components, in addition to exerting an adverse oxidation stress that leads to accumulation of the intracellular ROS. The MIC of chitosan (produced by SpsCDA) that inhibited spore germination of *F. oxysporum* was 1.56 mg/mL. Similar results on the inhibition of spore germination as an antifungal mechanism of chitosan against *Fusarium graminearum* have been reported (Luan et al. 2022; Deshaies et al. 2022). Similar studies demonstrated that chitin arrested the spore germination of *Phytophthora infestans* and *Alternaria alternata* (Huang et al. 2021; Zhang et al. 2021). These findings confirm previous reports demonstrating the remarkable inhibitory influence of chitosan on spore germination of *Ceratocystis fimbriata*, *Aspergillus ochraceus*, and *Fusarium solani* (Xing et al. 2018; Meng et al. 2020; Ghule et al. 2021). As spore germination is the first step in the development of fungi, the prevention of their germination could be an effective strategy to combat the widespread fungi (Abd El-Ghany et al. 2023a). To understand how chitosan inhibits spore germination, we investigated the potential ultrastructural alternations of spores upon exposure to chitosan by electron microscopy. TEM observation proved the damage of chitosan-treated spores with prominent ultrastructure alterations accompanied by the disintegration of membranes and the increased leakage of proteins and DNA verified that damage. This impairment is in harmony with that caused by exposure of *A. ochraceus* to chitosan (Meng et al. 2020). These results concord with the previously published observations depicting the ultrastructural alternations induced by exposure of *Fusarium sulphureum* and *Rhizoctonia solani* to chitosan (Li et al. 2009; Liu et al. 2012). It has been suggested that the electrostatic interactions between chitosan and the negatively charged phospholipids alter the fluidity of the plasma membrane and trigger membrane permeabilization (Palma-Guerrero et al. 2010). Furtheremore, it has been reported that chitosan may promote an osmotic imbalance that leads to disruction of the cell membrane allowing extravasation of the cytoplasmic materials (Feng et al. 2021; Oliveira et al. 2019). However, the exact mechanism that promotes these ultrastructural modifications has yet to be fully understood and requires further investigations for better understanding. In a recent study based on transcriptome analysis of chitosan-treated *P. infestans*, chitosan is thought to inhibit spore germination by affecting the metabolism of lipids, amino acids, and carbohydrates (Huang et al. 2021). Moreover, we demonstrated the chitosan-induced accumulation of ROS that might result in lipid peroxidation, an oxidative damage caused by targeting the membrane lipid (Hao et al. 2023). Also, excessive ROS induces inhibition and morphological damage to fungal spores (Ashraf et al. 2020) and plays a critical role in the process of nuclear damage-induced cell death (Pan et al. 2023). Thus, our findings suggest that chitosan can be used as an antifungal agent to protect plants from fungal phytopathogens. In conclusion, chitin was converted to chitosan by removing the acetyl groups with chitin deacetylase derived from *S. psychrophila* WP2. The cold-active enzyme accomplished the bioconversion process in 72 h at 15 °C with a yield of 69.2%. The produced moderate molecular weight chitosan (Mwt 224.7 kDa) with a degree of deacetylation of 78.1% had a CrI value of 18.75. Further, the biologically extracted chitosan arrested the spore germination of *F. oxysporum* with a MIC value of 1.56 mg/mL, and its proposed antifungal mechanism attributed to disruption of membrane permeability and integrity and exserting oxidative stress that is affirmed by accumulation of ROS. This work highlighted the merits of the cold-active chitin deacetylase from *S. psychrophila* for green conversion of chitin to bioactive chitosan.

## Data Availability

The essential data supporting the reported results are contained in this study. All other data is available upon request from the corresponding author.
